# Development of a polyamine gene expression score for predicting prognosis and treatment response in clear cell renal cell carcinoma

**DOI:** 10.3389/fimmu.2022.1048204

**Published:** 2022-11-25

**Authors:** Mei Chen, Zhenyu Nie, Denggao Huang, Yuanhui Gao, Hui Cao, Linlin Zheng, Shufang Zhang

**Affiliations:** Central Laboratory, Affiliated Haikou Hospital of Xiangya Medical College, Central South University, Haikou, China

**Keywords:** polyamine metabolism, clear cell renal cell carcinoma, immunotherapy, immune escape, multi-omics, prognosis

## Abstract

**Backgrounds:**

Polyamine metabolism (PM) is closely related to the tumor microenvironment (TME) and is involved in antitumor immunity. Clear cell renal cell carcinoma (ccRCC) not only has high immunogenicity but also has significant metabolic changes. However, the role of PM in the immune microenvironment of ccRCC remains unclear. This study aimed to reveal the prognostic value of PM-related genes (PMRGs) expression in ccRCC and their correlation with the TME.

**Methods:**

The expression levels PMRGs in different cells were characterized with single-cell sequencing analysis. The PMRG expression pattern of 777 ccRCC patients was evaluated based on PMRGs. Unsupervised clustering analysis was used in identifying PMRG expression subtypes, and Lasso regression analysis was used in developing polyamine gene expression score (PGES), which was validated in external and internal data sets. The predictive value of PGES for immunotherapy was validated in the IMvigor210 cohort. Multiple algorithms were used in analyzing the correlation between PGES and immune cells. The sensitivity of PGES to chemotherapeutic drugs was analyzed with the “pRRophetic” package. We validated the genes that develop PGES in tissue samples. Finally, weighted gene co-expression network analysis was used in identifying the key PMRGs closely related to ccRCC, and cell function experiments were carried out.

**Results:**

PMRGs were abundantly expressed on tumor cells, and PMRG expression was active in CD8^+^ T cells and fibroblasts. We identified three PMRG expression subtypes. Cancer and immune related pathways were active in PMRG expression cluster A, which had better prognosis. PGES exhibited excellent predictive value. The high-PGES group was characterized by high immune cell infiltration, high expression of T cell depletion markers, high tumor mutation burden and tumor immune dysfunction and exclusion, was insensitive to immunotherapy but sensitive to sunitinib, temsirolimus, and rapamycin, and had poor prognosis. Spermidine synthetase (SRM) has been identified as a key gene and is highly expressed in ccRCC at RNA and protein levels. SRM knockdown can inhibit ccRCC cell proliferation, migration, and invasion.

**Conclusions:**

We revealed the biological characteristics of PMRG expression subtypes and developed PGES to accurately predict the prognosis of patients and response to immunotherapy.

## Introduction

Renal carcinoma is a common malignant tumor, 80% of which is clear cell renal cell carcinoma (ccRCC) (1). Immune checkpoint and tyrosine kinase inhibitors can be regarded as systemic therapies for patients with advanced or metastatic ccRCC (2). However, only 42% of patients respond to treatment because of tumor heterogeneity, and the median progression free survival is 11.6 months (3). No effective biomarker for predicting the response of patients with ccRCC patients to immunotherapy is available. Therefore, effective biomarkers or tools are urgently needed to guide precision therapy. ccRCC is highly immunogenic, and understanding the heterogeneity of the tumor immune microenvironment can provide targeted immunotherapy to patients.

Polyamine metabolism (PM) is closely related to the tumor microenvironment (TME) and is involved in antitumor immunity (4, 5). Polyamines mainly include spermine, spermidine, and putrescine (6). The regulation of polyamines is a rigorous process, including polyamine synthesis, transport, and catabolism, and is widely involved in cell proliferation, apoptosis, and gene regulation. ODC1 and AMD1 encode the rate-limiting enzymes ornithine decarboxylase and S-adenosine methionine decarboxylase for polyamine synthesis, respectively. Spermine synthetase (SMS) and spermidine synthetase (SRM) are involved in the synthesis of spermine and spermidine, respectively. Spermine oxidase (SMOX) participates in the decomposition of spermine. Spermine/spermidine N^1^-acetyltransferase (SSAT) acetylates the N^1^ position of spermine or spermidine, which can be oxidized back to the preceding polyamine in the biosynthetic pathway by peroxisome acetylpolyamine oxidase (PAOX). OAZ, as ornithine decarboxylase (ODC) antizyme, includes OAZ1, OAZ2, and OAZ3 (7). AZIN, as ODC anti-enzyme inhibitor, includes AZIN1 and AZIN2 (8). OAZ1 can inhibit polyamine production and oral cancer cell proliferation (9). ATP13A2 plays a transport role in polyamine synthesis, is located in late endolysosomes, promotes the cellular uptake of polyamines through endocytosis, and transports them into the cytoplasm (10). ATP13A3 transports vesicular polyamines, is more active against putrescine, and localizes to early and circulating endosomes (11). Polyamine transport in pancreatic cancer is mediated by ATP13A3 (12). SLC18B1 is responsible for the storage and release of polyamines, which is a polyamine vesicle transporter (13, 14). Recently, it has been reported that the formation of tumor immunosuppression can be caused by the increase of polyamine level (15, 16). Arginine is the main donor for polyamine synthesis in T cells, glutamine is a minor donor, and decrease in polyamine level can inhibit T cell proliferation (17). Arginine promotes T-cell proliferation and activation through its metabolism to ornithine *via* arginase (18). ODC can induce the polarization of M2 macrophages (19).Polyamines metabolic enzymes are frequently dysregulated in tumor cells (20–23). AMD1 and SMOX are highly expressed in cancer, which is related to poor prognosis of patients and plays a carcinogenic role (24–27). ARG1 promotes ovarian cancer progression by suppressing T cell immune responses (28). ATP13A2 is a prognostic marker and potential therapeutic target of colon cancer, which can inhibit tumor occurrence by blocking autophagic flux (29). Deletion of autophagy gene can regulate circulating arginine and inhibit tumor progression (30). Targeting PMRGs is a promising target for cancer therapy (31). The immune modulatory vaccine based on ARG1 can induce anti-tumor immunity and has synergistic anti-tumor effect with Anti-PD-1 checkpoint blockade (32). OAZ1 and SAT1 can increase the sensitivity of non-small cell lung cancer and bladder cancer cells to cisplatin, respectively (33, 34). Metformin can reduce the expression of ARG1 and ODC, reduce the formation of putrescine and inhibit the progression of colorectal cancer (35). Inhibitors targeting polyamine metabolic enzymes have become new therapeutic strategies, such as ARG1 inhibitor CB-1158 (36, 37), ODC multipurpose inhibitor (38), SMOX inhibitor (39). Polyamine metabolism-related genes (PMRGs) play an important role in the immune microenvironment and can be used as immunotherapy targets (40–42). A significant change in ccRCC is metabolic change, and the expression of metabolism-related genes is dysregulated in ccRCC (43, 44). Studies have shown that polyamine levels are positively correlated with ccRCC invasiveness and are involved in the progression of cancer (45). Although targeting PM in ccRCC is a promising therapeutic strategy, how PM affects the TME of ccRCC, and its role in immunotherapy is still unclear.

In this study, we evaluated the expression profile of PMRGs on ccRCC, described the expression of PMRGs on TME from the single-cell sequencing level. We developed three PMRG expression subtypes, identified two gene clusters, and established the polyamine gene expression score (PGES) to predict the prognoses of ccRCC patients and response to immunotherapy. We analyzed the correlation between the PGES and immune cells with different calculation methods. Finally, the effect of SRM, the key PMRG, on the biological function of ccRCC cells was detected, and the expression levels of genes used in developing the PGES were validated in clinical tissues.

## Methods

### Data source and preprocessing

The expression data (fragments per kilobase million, FPKM) and clinical data of the ccRCC cohort were downloaded from The Cancer Genome Atlas (TCGA) database (https://portal.gdc.cancer.gov/), and the expression data and clinical data of the E-MTAB-1980 data set were downloaded from the ArrayExpress database (https://www.ebi.ac.uk/arrayexpress/). The FPKM was converted into transcripts per kilobase million (TPM). Batch effects were corrected with the “Combat” algorithm. A total of 777 patients with survival times were included for analysis when the two datasets were merged. External validation cohort (GSE22541) and single-cell data set (GSE171306) were obtained from the GEO (https://www.ncbi.nlm.nih.gov/geo/), respectively. The “FeaturePlot” and “VlnPlot” functions in the “seurat” package were used in describing the gene expression patterns and violin plot in each subgroup, respectively. Relative data and information of immunotherapy cohort (IMvigor210) was download from website (http://research-pub.gene.com/IMvigor210CoreBiologies). Clinical Proteomic Tumor Analysis Consortium (CPTAC, https://proteomics.cancer.gov/programs/cptac) provide proteomic data for analysis. Abbreviations from this study are summarized in **Supplementary Table S1**.

### Unsupervised cluster analysis of polyamine metabolism

We collected 16 genes involved in PM from the literature (5) (**Supplementary Table S2**). The consistency clustering analysis was carried out with the “consensusclusterplus” package, and the clustering effect was considered the best when k = 3. The three subtypes were analyzed by principal component analysis (PCA) and visualized by “scatterplot3d” package. Then gene set variation analysis (GSVA) was used in analyzing the differences of biological characteristics among the subtypes and performing with the “gsva” package. “c2.cp.kegg.v7.5.1.symbols.gmt” as hallmark gene set was downloaded from the MSigDB database (https://www.gsea-msigdb.org/gsea/msigdb).

### Enrichment analysis of differentially expressed genes (DEGs) and identification of gene clusters

The “limma” package was used in analyzing DEGs among PMRG expression subtypes. The enrichment of DEGs is first analyzed by GO and then by KEGG. The analyses were carried out by the “clusterProfiler” package. Prognostic DEGs were screened by univariate Cox regression and performed unsupervised clustering to produce gene subtypes.

### Development and validation of PGES

The “caret” package was used in randomly dividing patients into training group (n = 317) and testing group (n = 316) in a ratio of 1:1. PGES was developed by Lasso Cox regression analysis of DEGs with prognostic value.


$$PGES=\sum_{i=1}^{n}Coef_{i}\times Exp_{i}$$

Coef in the formula represents risk coefficient, and Exp represents gene expression. The patients were divided into high- and low-PGES groups according to the median of the scores of the training group. The “timeROC” package was used in drawing receiver operator curves (ROC) curve. External validation was carried in E-MTAB-1980 and GSE22541 data sets. The “rms” package was used in constructing a nomogram for visualizing the PGES.

### Functional differences and immune infiltration characteristics of high and low PGES

GSEA was performed with the “clusterprofiler” package. The ssGSEA algorithm was used in evaluating the degree of tumor immune infiltration and analyzing the differences in immune cells and immune functions between high and low PGES (46). The The proportion of different types of cells was calculated by the CIBERSORT algorithm (47). Immune and stromal scores were calculated by the ESTIMATE algorithm (48).

### Evaluation of the role of PGES in immunotherapy and chemotherapy

Mutation data were analyzed and visualized with the “maftools” package, the tumor mutation burden (TMB) of each patient in the TCGA cohort was calculated, and the difference in TMB between high and low PGES was analyzed. The tumor immune dysfunction and exclusion (TIDE) score of ccRCC was downloaded from the TIDE database (http://tide.dfci.harvard.edu/), and the TIDE score between high and low PGES was analyzed. The “pRRophetic” package was used in predicting the sensitivity of different PGES groups to therapeutic drugs.

### Sample collection

The paired cancer and paracancerous tissues of 12 ccRCC patients were collected from the hospital. The samples were stored at −80°C. The study was approved by the ethics committee of Affiliated Haikou Hospital of Xiangya Medical College, Central South University. Patients’ informed consent was obtained before collection. The reagents, procedures, and calculation methods used in quantitative real-time PCR (qRT-PCR) were consistent with those in our previous study (49). Amplification reactions were performed on QuantStudio 5 instrument (Applied Biosystems). The primer sequences designed for amplification are shown in **Supplementary Table S3**.

### Identification of key PMRGs

To identify the key PMRGs closely related to ccRCC, a weighted gene coexpression network analysis (WGCNA) was constructed from the TCGA expression profile with the “WGCNA” package. We screened the key genes by taking the intersection of genes in the module positively related to tumor and PMRGs.

### Construction of stable knockdown cells

ccRCC cells (786-0 and 769-P) were purchased from the China Centre for Type Culture Collection (CCTCC, Wuhan,China). Lentivirus was purchased from Genechem (Shanghai, China), and the transfection protocol followed the manufacturer’s instructions. Puromycin was used in screening stable knockdown cells. Knockdown efficiency was measured by qRT-PCR.

### *In vitro* cellular functional assays

For CCK-8 assays, 786-0 (1000 cells), and 769-P (2000 cells) were cultured on 96-well plates. On days 0, 1, 2, 3, and 4, approximately 10 μL of CCK-8 (Dojindo, Japan) was added to each well, incubated at 37°C for 1 h, and then detected with 450 nm absorbance. Cells were exposed to different concentrations of sunitinib and sorafenib. After 48h of treatment, OD values were detected. Sorafenib and sunitinib were purchased from MedChemExpress (Monmouth Junction, NJ, United States). For the colony formation assays, 786-0 (1000 cells) and 769-P (2000 cells) were cultured on six-well plates for 14 days, fixed with methanol, and stained with 0.1% crystal violet. For the Transwell assay, 786-0 (1 × 10^4)^ and 769-P (5 × 10^4)^ diluted in serum-free medium was added to the upper chamber. For the scratch assays, 786-0 (5 × 10^5^ cells) and 769-P (7 × 10^5^ cells) were cultured on six-well plates. On the second day when cells were full, lines were drawn in the central area with 200ul gun tip, washed twice with PBS, and then replaced with serum-free medium. Cells were observed and pictures were taken at 0, 24, and 48h under microscope. The detailed experimental protocols are described in previous reseesrch (50).

### Statistical analysis

Statistical analysis was performed in R software (version 4.1.2) and Graphpad Prism (version 8.0.2). The comparison between the two groups was performed by T test. The Kaplan–Meier method was performed for prognosis among groups, and log rank test was used in evaluating statistical difference. Univariate COx regression analysis was used in screening prognostic genes. Pearson test was used for correlation analysis. *P<* 0.05 was considered statistically significant.

## Results

### Multi-omics analysis of the expression patterns of PMRGs in ccRCC

**Figure 1:**
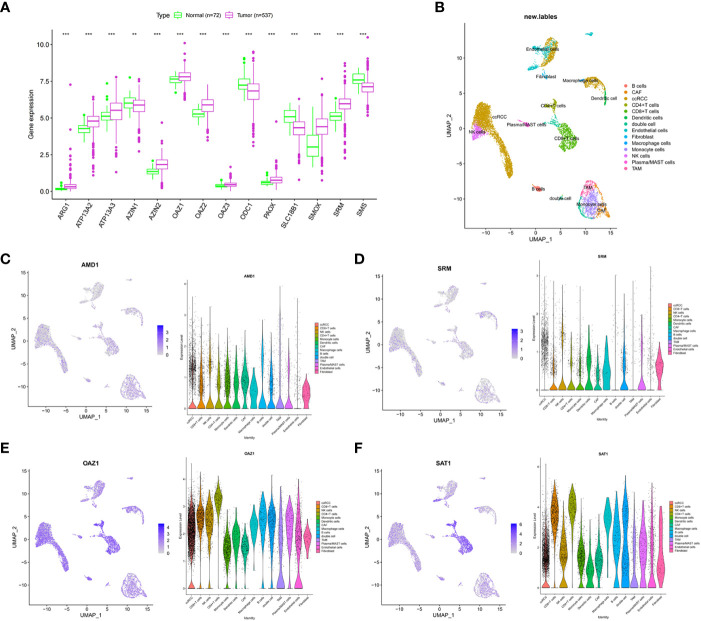
Multi-omics analysis of PMRGs in ccRCC. **(A)** Expression levels of PMRGs in the TCGA-ccRCC cohort. **(B)** UMAP plot of single cells in GSE171306 data set. **(C–F)** Distribution and expression patterns of AMD1, SRM, OAZ1, and SAT1 in single cells. PMRGs, polyamine metabolism-related genes; ccRCC, clear cell renal cell carcinoma. **p < 0.01, ***p < 0.001.

In the TCGA-ccRCC cohort, ARG1, ATP13A2, ATP13A3, AZIN2, OAZ1, OAZ2, OAZ3, PAOX, SMOX, and SRM were highly expressed in cancer tissues, whereas AZIN1, ODC1, SLC18B1, and SMS had low expression levels in cancer tissues, compared with corresponding normal tissues (**Figure 1A**). The high expression levels of AMD1, AZIN1, ODC1, SLC18B1 are related to the good prognosis of ccRCC patients (**Supplementary Figure 1**). Proteomic data showed that ARG1, ATP13A3, and SRM were highly expressed in cancer tissues, whereas PAOX and SMS had low expression (**Supplementary Table S4**). The cohort analyzed did not include data for the other polyamine metabolism-related proteins of interest. We further used single-cell sequencing data to analyze the expression level of PMRGs in ccRCC at the single-cell level. In the GSE171306 dataset, we combined two ccRCC samples for analysis and obtained a total of 18,462 cells after quality control. The annotated cell clusters included B cells, CAF, ccRCC, CD4^+^T cells, CD8^+^T cells, dendritic cells, double cells, endothelial cells, fibroblast, macrophage cells, monocyte cells, NK cells, plasma/MAST cells, and TAM (**Figure 1B**). PMRGs were expressed explicitly on ccRCC (**Figures 1C–F**; **Supplementary Figure 2**). In addition, we found that PMRGs were expressed on immune cells, especially CD8^+^ T cells and fibroblasts, suggesting that these cells may have relatively active PMRG expression. Among them, both OAZ1 and SAT1 were highly expressed in all cell types (**Figures 1E, F**). These results suggested that PMRGs play an important role in ccRCC and were closely related to immunity.

### Identification of polyamine clusters and functional analyses

**Figure 2:**
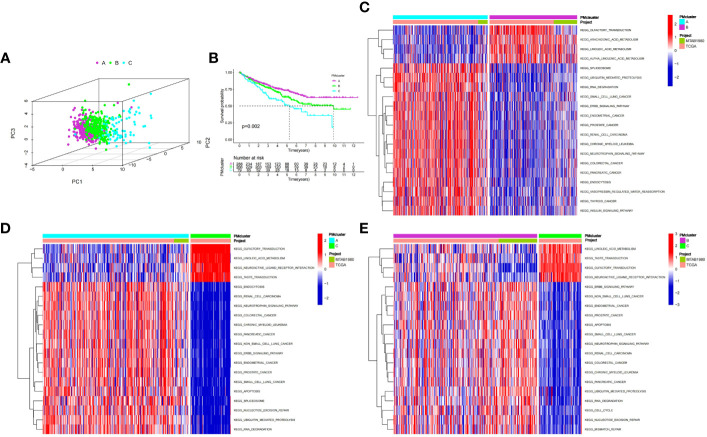
Identification and biological characteristics of PMRG expression cluster. **(A)** Three-dimensional PCA showed the difference of PMRG expression i clusters A, B and C. **(B)** Kaplan–Meier curves of survival differences among the three PMRG expression clusters. **(C–E)** Biological pathways enriched among different PMRG expression clusters. PMRG, polyamine metabolism-related gene.

We used an unsupervised clustering method to classify patients into three PMRG expression clusters: A (n = 288), B (n = 266), and C (n = 79) according to the expression values of 16 PMRGs. PCA indicated significant differences among the three subtypes (**Figure 2A**). PMRG expression cluster A had the longest survival time (*P* = 0.002, **Figure 2B**). The expression of PMRGs was highest in PMRG expression cluster A (**Supplementary Figure 3**). Moreover, PMRG expression cluster A had the highest number of activation pathways and was mainly enriched in cancer and immune-related pathways, such as renal cell carcinoma, ERBB signaling pathway, and endocytosis. Compared with PMRG expression cluster A and B, cluster C was mainly enriched in metabolic pathways, such as linoleic acid metabolism (**Figures 2C–E**). These results indicated that PMRG expression is closely related to cancer and immunity.

### Development of PMRG expression gene cluster

**Figure 3:**
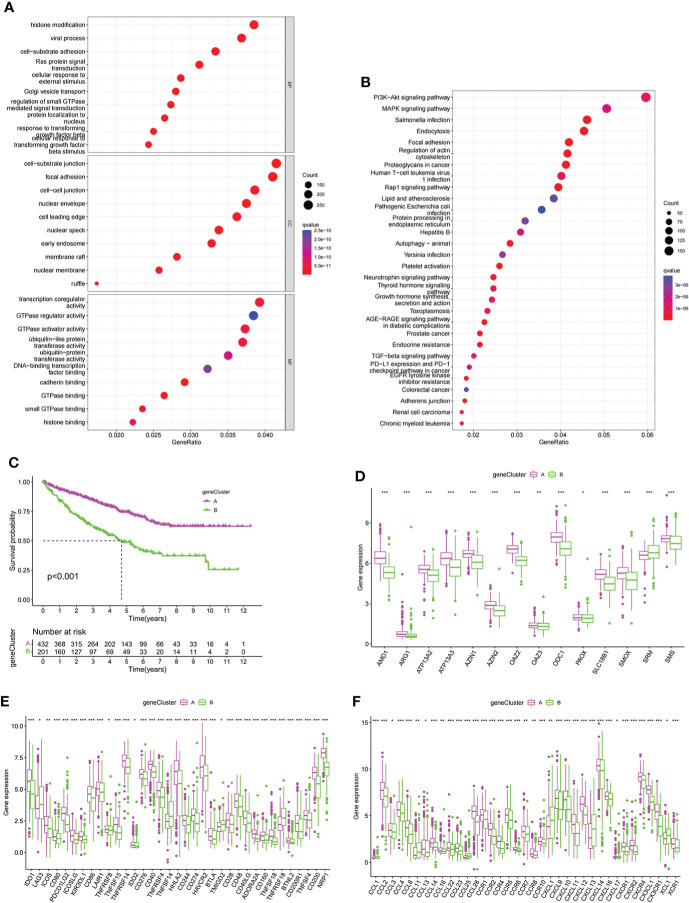
Development of gene cluster. **(A, B)** GO and KEGG analyses of DEGs. **(C)** K–M curve of survival difference of gene clusters. **(D)** Expression levels of PMRGs among different gene clusters. **(E)** Differences in immune checkpoint expression among gene clusters. **(F)** Differences in chemokine expression among gene clusters. PM, polyamine metabolism; PMRGs, polyamine metabolism-related genes. *p < 0.05, **p < 0.01, ***p < 0.001.

To further explore the potential biological functions of PMRG expression clusters, we identified 692 DEGs among the three PMRG expression clusters and performed GO and KEGG enrichment analysis on the DEGs. GO analysis showed that DEGs were significantly enriched in signal transduction and proliferation. Biological processes mainly included Ras protein signal transduction. Cell components mainly included cell–substrate junction and focal adhesion (**Figure 3A**). Molecular functions mainly included transcription coregulator activity and GTPase regulator activity. KEGG analysis found that DEGs were enriched in cancer metabolism and immune-related pathways, such as the PI3K-Akt signaling pathway, PD-1 checkpoint pathway in cancer (**Figure 3B**). These results indicated that PMRG expression plays a key role in cancer and immune regulation. Univariate Cox regression analysis was performed on DEGs, and 651 genes with prognostic significance were obtained. We performed unsupervised clustering on these DEGs with prognostic value, and the algorithm was optimal when the DEGs were divided into two clusters. Gene cluster A had a better survival time (**Figure 3C**). PMRGs showed significant differences, and the expression level of cluster A was the highest (**Figure 3D**). Of the 35 differentially expressed immune checkpoints, 31 were highly expressed in gene cluster A (**Figure 3E**). Thirty of the thirty-seven differentially expressed chemokines were highly expressed in gene cluster A (**Figure 3F**).

### PGES development and validation

**Figure 4:**
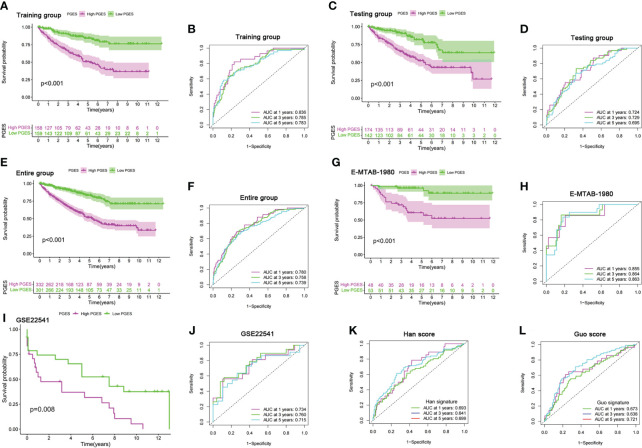
Prognostic value of the PGES. **(A, B)** K-M curve of survival difference and predictive accuracy of PGES in the training group. **(C, D)** K–M curve of survival difference and predictive accuracy of PGES in the testing group. **(E, F)** K–M curve of survival difference and predictive accuracy of PGES in the entire group. **(G, H)** K–M and ROC curves of PGES predicting survival in E-MTAB-1980 dataset. **(I, J)** K–M and ROC curves of PGES predicting survival in GSE22541 dataset. **(K, L)** Predictive accuracy of PGES in other studies. PGES, polyamine gene expression score.

LASSO regression analysis was performed on prognostic DEGs to obtain the key genes used in developing the PGES. PGES = (−0.136 × expression of EMX2) + (−0.235 × expression of EDA) + (−0.212 × expression of OPCML) + (−0.323 × expression of SEMA3G)+ (−0.217 × expression of ENPP5) + (0.287 × expression of PCDHGC3). In the training group, the survival times of patients in the high-PGES group were shorter than those in the low-PGES group (**Figure 4A**). ROC curves showed that AUCs at 1, 3, and 5 years were 0.836, 0.785, and 0783, respectively (**Figure 4B**).

To verify the applicability of PGES, we conducted internal and external validation. In the testing and entire groups, the prognoses of low-PGES patients was better, and the PGES had good predictive power (**Figures 4C–F**). In the external validation of the E-MTAB-1980 and GSE22541 datasets, the survival times of low-PGES patients were longer (**Figures 4G, I**), and the ROC curves showed that the AUCs of 1, 3, and 5 years were greater than 0.7, indicating that the PGES had good predictive power and applicability (**Figures 4H, J**). To visualize the model, we combined risk and clinicopathological variables to construct a nomogram for predicting the survival of patients at 1, 3, and 5 years (**Supplementary Figure 4A**). The ROC curve suggested that the nomogram had good prediction accuracy (**Supplementary Figure 4B**).

Compared with the score constructed by Han (51) and Guo (52) in ccRCC, our score had higher diagnostic efficacy and better prediction power (**Figures 4K, L**). Furthermore, we found that cluster A had the lowest score in the PMRG expression cluster and gene cluster (**Supplementary Figures 4C, D**), which may explain the better prognosis in cluster A.

### Functional differences and immune infiltration characteristics of patients in high- and low-PGES groups

**Figure 5:**
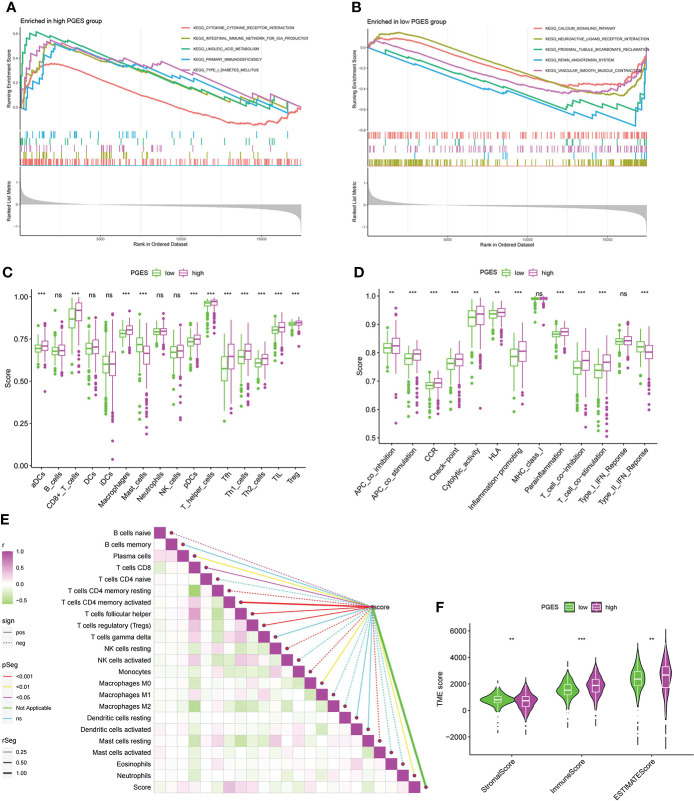
Functional enrichment analyses and immune infiltration characterization of different PGES groups. **(A, B)** Pathways enriched at high and low PGES. **(C, D)** Differences in immune cells and immune function among different PGES groups based on ssGSEA algorithm. **(E)** Correlation analysis between PGES and immune cells based on the CIBERSORT algorithm. **(F)** The stromal, immune, and estimate scores of the high- and low-PGES groups were evaluated by the estimate algorithm. PGES, polyamine gene expression score. **p < 0.01, ***p < 0.001 and ns means no significance.

To analyze the functional differences between high- and low-PGES groups, we performed GSEA analysis and found that immune and metabolic pathways were significantly enriched in the high-PGES groups, such as cytokine cytokine receptor interactin, primary immunodeficiency, and linoleic acid metabolism (**Figure 5A**). Calcium signaling pathway, neuroactive ligand receptor interaction, and proximal tubule bicarbonate reclamation were significantly enriched in the low-PGES groups (**Figure 5B**).

Then immune cell infiltration in the high- and low-PGES groups was calculated by ssGSEA, CIBERSORT, and ESTIMATE algorithms. The results of the ssGSEA algorithm showed that T cells had higher enrichment scores in the high-PGES groups, such as CD8^+^ T, T helper, Tfh, Th1, and Th2 cells (**Figures 5C, D**). The results of the CIBERSORT algorithm showed that the PGES was positively correlated with plasma cells, CD8^+^T cells, CD4 memory-activated T cells, follicular helper T cells, and regulatory T cells (Tregs), M0 macrophages, and neutrophils. However, PGES was negatively correlated with resting immune cells, such as naïve B cells, CD4 memory resting T cells, resting NK cells, and resting mast cells (**Figure 5E**). Based on the ESTIMATE algorithm calculating the immune and stromal scores to predict the infiltration of nontumor cells, we found that the stromal score was higher in the low-PGES group, and the immune and estimate scores were higher in the high-PGES group (**Figure 5F**). These results indicated that the high-PGES group had a higher degree of immune cell infiltration.

### Role of score in immunotherapy and chemotherapy

**Figure 6:**
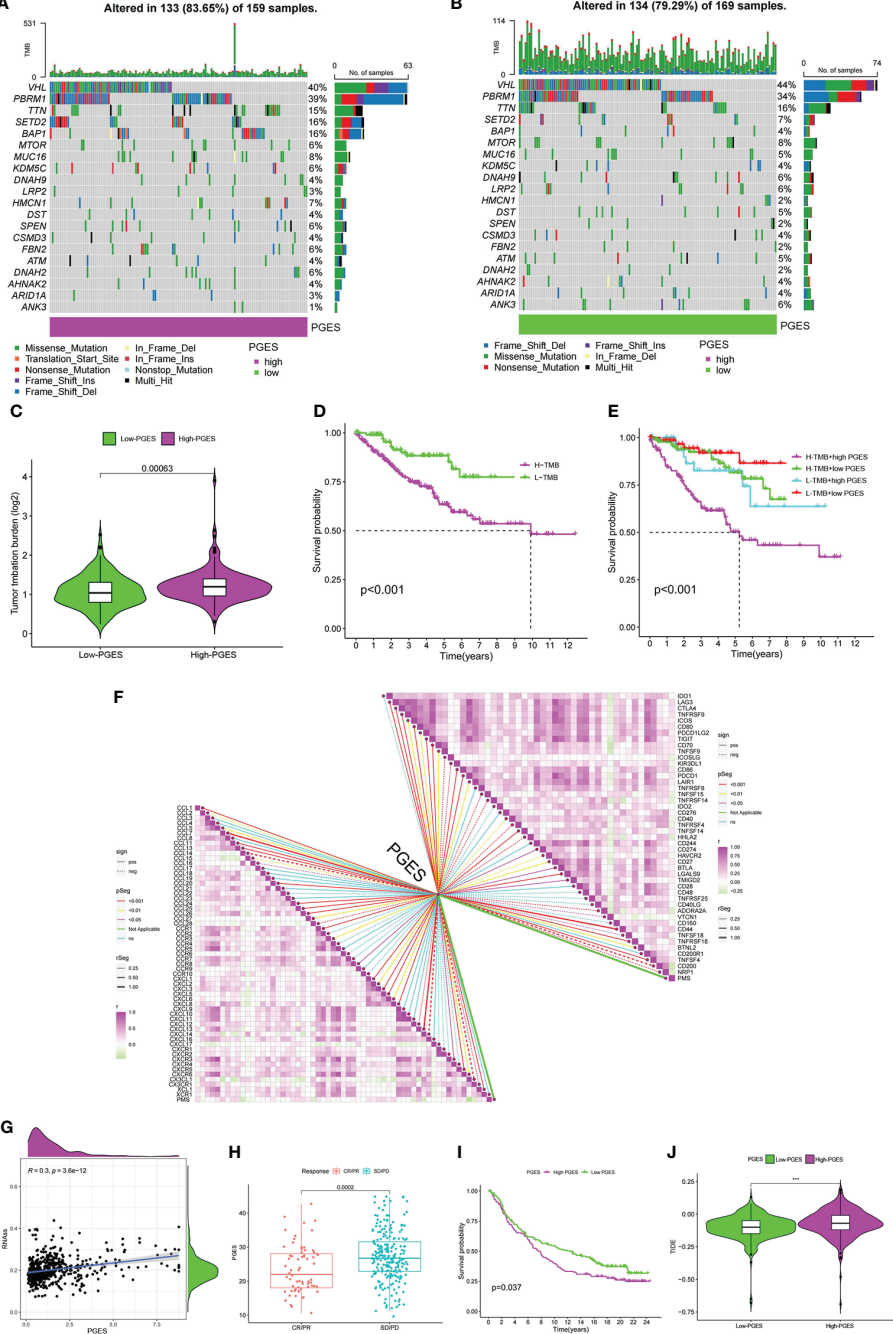
Predictive value of PGES on ccRCC immunotherapy. **(A, B)** The waterfall plot shows the mutation frequency of the high- and low-PGES groups. **(C)** TMB differences between high and low PGES. **(D)** K–M curve of survival difference between high and low TMB. **(E)** K–M curve of survival difference stratified by TMB and PGES. **(F)** Correlation analysis of PGES with immune checkpoint and chemokines. **(G)** Correlation analysis between PGES and stemness indices. **(H)** Boxplot shows PGES difference between CR/PR and SD/PD. **(I)** K–M curve of difference in overall survival between high and low PGES. **(J)** Difference in TIDE between high and low PGES. PGES, polyamine gene expression score; ccRCC, clear cell renal cell carcinoma. ***p < 0.001.

The high-PGES group had more mutations, and PBRM1, SETD2, and BAP1 were higher in the high-PGES group (**Figures 6A, B**). The TMB in the high-PGES group was higher, indicating that increase in neoantigens and active immunity, which is consistent with the characteristics of immune infiltration (**Figure 6C**).

The prognosis of the high TMB group was worse than that of the low-TMB group (**Figure 6D**). Among the combinations of TMB and PGES, the prognosis of high-PGES patients in the high TMB group was the worst (**Figure 6E**). A total of 47 immune checkpoints were analyzed, 36 of which were correlated with PGES, which was positively correlated with T cell depletion markers PDCD1, CTLA-4, TIGIT, and LAG3. Of the 32 differentially expressed chemokines, 24 were positively correlated with PGES (**Figure 6F**). PGES was positively correlated with stemness indices (**Figure 6G**), indicating that high-PGES patients had lower degrees of cell differentiation and higher malignancy, which explains the poor prognoses of the high-PGES patients. In the IMvigor210 cohort, the SD/PD group (nonresponders) had a higher PGES (**Figure 6H**), and the prognoses of patients in the high-PGES group were poor (**Figure 6I**). The TIDE of patients in the high-PGES group was higher (**Figure 6J**). Although the high-PGES patients had higher degrees of immune cell infiltration, they might not be sensitive to immunotherapy because of immune escape. These results indicated that PGES can effectively predict the response of ccRCC on immunotherapy.

**Figure 7:**
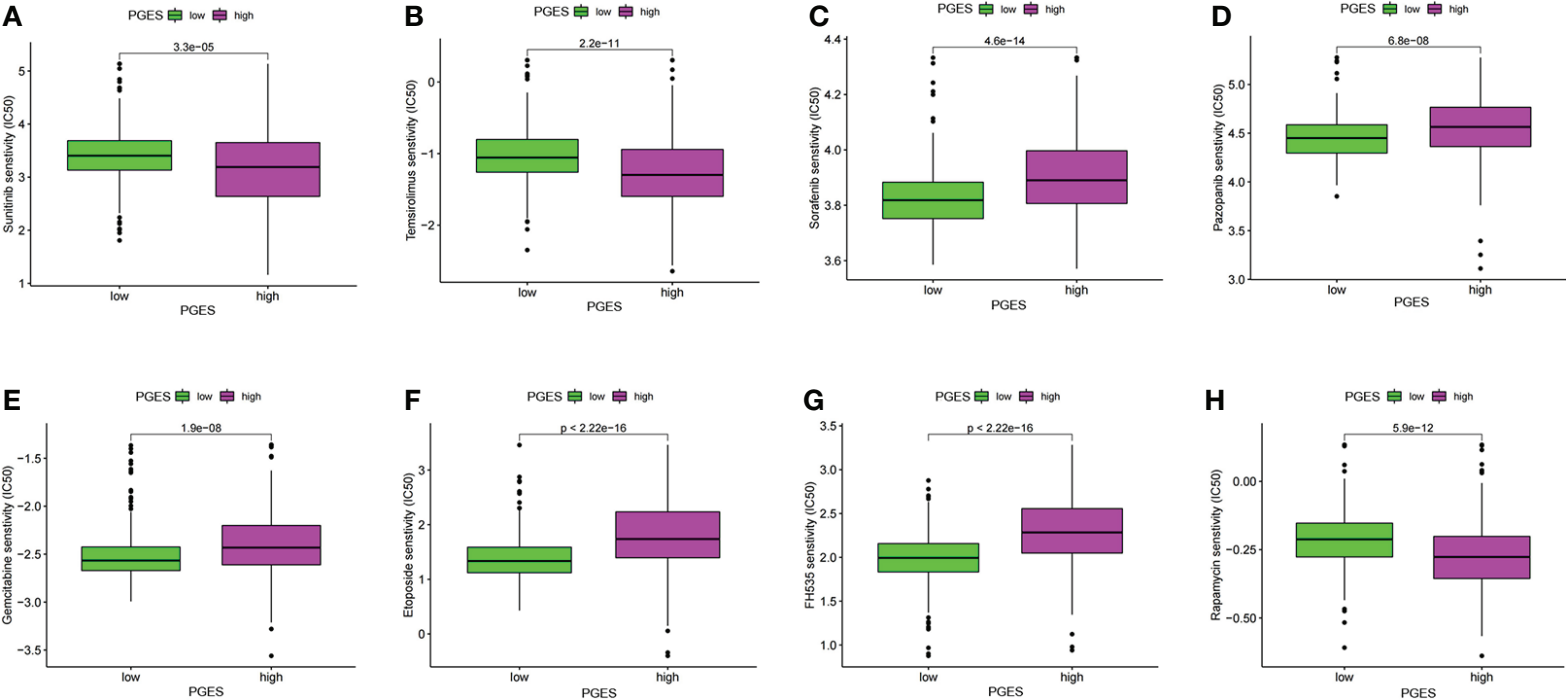
PGES and drug sensitivity analyses. **(A–H)** The drug sensitivity difference of sunitinib, temsirolimus, sorafenib, pazopanib, gemcitabine, etoposide, FH535, rapamycin in high and low PGES. PGES, polyamine gene expression score.

The “pRRophetic” package was used in predicting the sensitivity of different PGES groups to therapeutic drugs. Among the common chemotherapeutic drugs in ccRCC, patients with high PGES were sensitive to sunitinib and temsirolimus (**Figures 7A, B**), whereas patients with low PGES were more sensitive to sorafenib, pazopanib, and gemcitabine (**Figures 7C–E**). In addition, we also found that targeting the cell cycle (etoposide) and Wnt/β-catenin pathway (FH535) was more effective in patients with low PGES (**Figures 7F, G**), whereas immunosuppressants (rapamycin) were more effective in patients with high PGES (**Figure 7H**).

### Validation in genes in tissue samples

**Figure 8:**
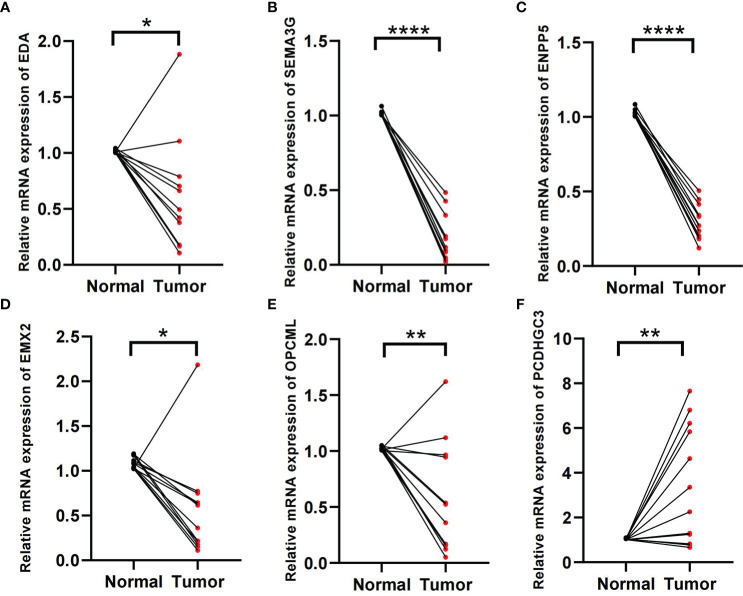
Validation of tissue samples. **(A–F)** Expression of EDA, SEMA3G, ENPP5, EMX2, OPCM, and PCDHGC3 in ccRCC cancer and normal tissues. *p < 0.05, **p < 0.01, ****p < 0.0001.

In the formula, EDA, SEMA3G, ENPP5, EMX2, and OPCML were favorable factors, whereas PCDHGC3 was a risk factor. We verified in the tissue samples that EDA, SEMA3G, ENPP5, EMX2, and OPCML had low expression levels in the ccRCC tissues (**Figures 8A–E**). By contrast, PCDHGC3 expression increased in the cancer tissues (**Figure 8F**).

### Screening of key PMRGs in ccRCC and its potential biological functions

**Figure 9:**
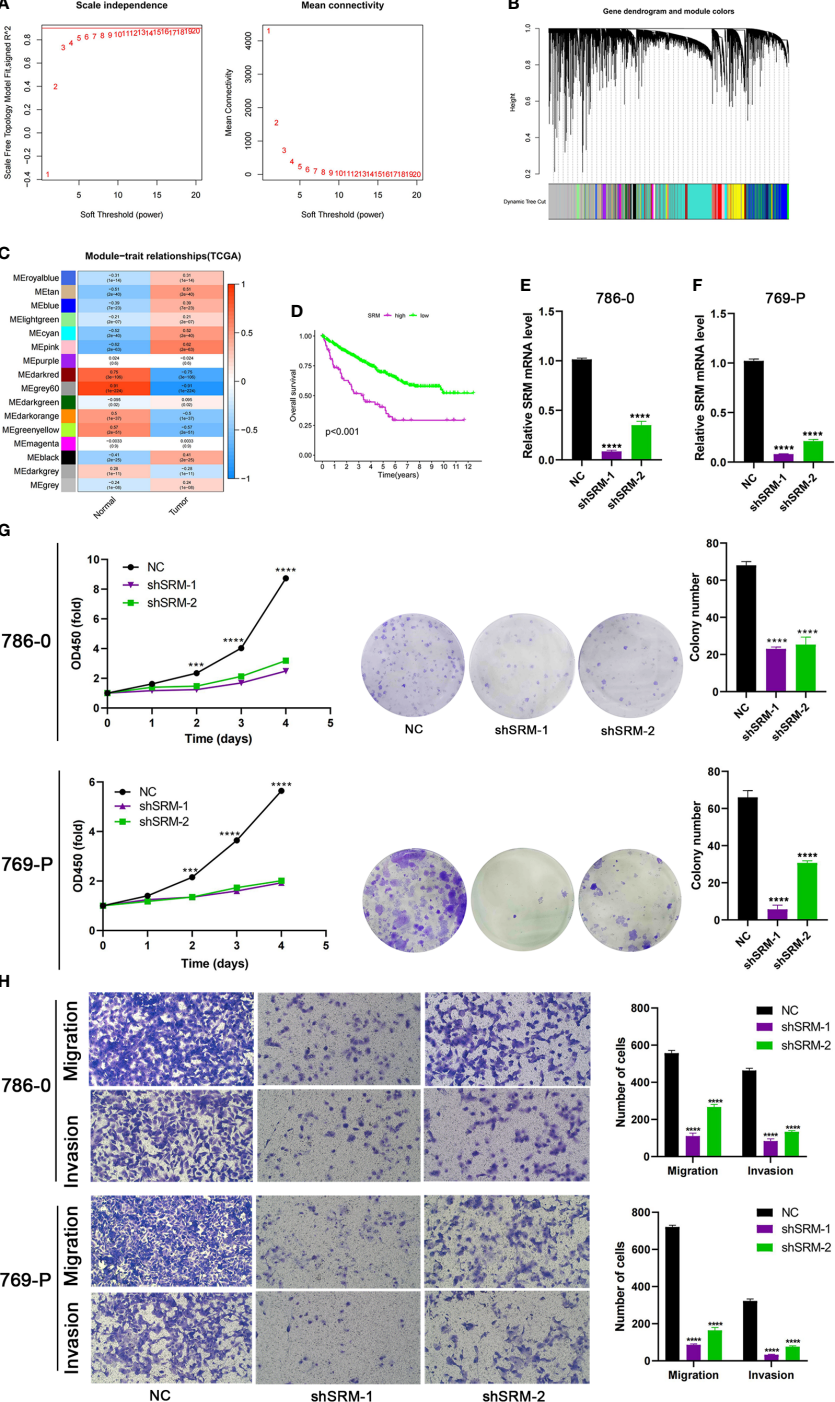
Identification of key PMRGs in ccRCC and *in vitro* cell function experiments. **(A)** Screening of soft threshold. **(B)** Hierarchical clustering dendrogram of 16 modules. **(C)** Heatmap of correlations between modules and tumor. **(D)** K–M curves of SRM in protein levels. **(E–F)** Knockdown efficiency of SRM in 786-0 and 769-P. **(G)** Effects of SRM knockdown on CCK8 and clone formation ability of ccRCC cells. **(H)** Transwell was used to detect the effect of SRM on the migration and invasion ability of 786-0 and 769-P. PMRGs, polyamine metabolism-related genes; ccRCC, clear cell renal cell carcinoma. ***p < 0.001, ****p < 0.0001.

**Figure 10:**
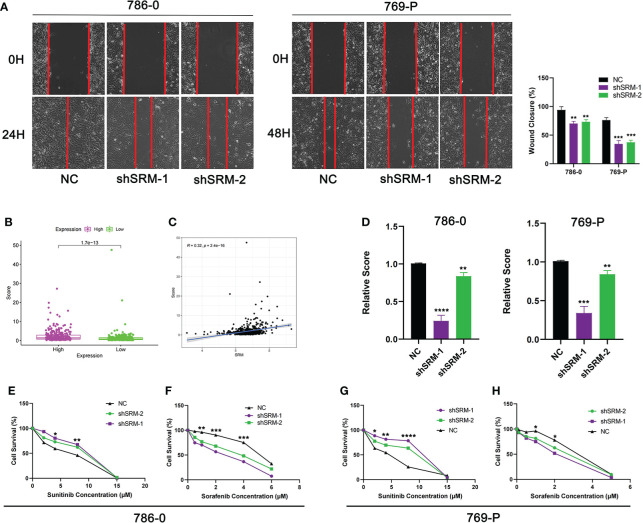
Validation of treatment response *in vitro*. **(A)** Scratch assay was used to detect the effect of SRM on the migration ability of 786-0 and 769-P. **(B)** Score difference between high and low SRM expression groups. **(C)** Correlation analysis between score and SRM expression. **(D)** Differences in scores between shSRM and negative control cell lines. **(E–F)** Effect of knockdown of SRM on 786-0 cell survival after treatment with sunitinib and sorafenib. **(G–H)** Effect of knockdown of SRM on 769-P cell survival after treatment with sunitinib and sorafenib. *p < 0.05, **p < 0.01, ***p < 0.001, ****p < 0.0001.

To further explore the role of PMRG expression in ccRCC, we used WGCNA to identify PMRGs closely related to ccRCC. When the soft threshold was 11, the fitting index (R^2^) and average connectivity of the scale-free topology model can reach a stable state (**Figure 9A**). A total of 16 modules were obtained (**Figure 9B**), we extracted genes from tan, cyan, and pink modules which were most associated with tumors (correlation coefficient > 0.5, **Figure 9C**). Then, we intersected these genes with the PMRGs. Finally, SRM was identified as the key PMRGs in ccRCC. Patients with high SRM expression at the protein level were associated with poor prognosis (**Figure 9D**). To explore the effect of SRM on the biological function of ccRCC cells, 786-0 and 769-P were selected for cell experiments. We transfected ccRCC cells with the lentivirus and detected interference efficiency by qRT-PCR. The lentivirus significantly interfered with the expression of SRM in the 786-0 and 769-P cells (**Figures 9E, F**). CCK8 and colony formation assays showed that the knockdown of SRM can significantly inhibit ccRCC cell proliferation (**Figure 9G**). Transwell assay showed that the SRM knockdown can significantly inhibit cell migration and invasion (**Figure 9H**). Scratch assay showed that knockdown of SRM could inhibit the migration of ccRCC cells (**Figure 10A**). In addition, we divided the patients into high expression and low expression groups based on the median SRM expression. Compared with the low expression group, the score of the high expression group was higher (**Figure 10B**). Correlation analysis showed that score was positively correlated with SRM expression (**Figure 10C**). qRT-PCR result showed that the score decreased after knockdown of SRM (**Figure 10D**). After treatment of ccRCC cells with sunitinib, knockdown of SRM increased cell survival, suggesting that knockdown of SRM could increase resistance to sunitinib (**Figures 10E, G**). After treatment of cells with sorafenib, knockdown of SRM reduced cell viability, suggesting that knockdown of SRM could increase the sensitivity of cells to sorafenib (**Figures 10F, H**).

## Discussion

PM in tumor cells is usually abnormal, and the occurrence of tumors is closely related to the abnormality of many enzymes in the process of PM (53–57). Recent studies have found that polyamines can regulate T cell differentiation and activate B cells, but their regulatory role in other immune cell types still needs further research (5). There is a complex cross talk mechanism between PM and TME. PM can become a potential target for improving tumor immunotherapy. However, PM in ccRCC TME has not been explored.

In this study, we analyzed the expression of PMRGs in ccRCC at the transcriptome and single-cell levels. PMRGs had higher expression levels in CD8^+^ T cells and fibroblasts. Polyamines regulate the differentiation of T cells (58). Chemokines secreted by fibroblasts can repel effector T cells while recruiting immunosuppressive cells (59). Fibroblasts are associated with metastasis of cancer patients and are a risk factor (60). We identified 3 PMRG expression subtypes based on 16 PMRGs. Significant differences in prognosis and biological pathways were found among subtypes. The prognosis of PMRG expression cluster A was better, and cancer and immune-related pathways were significantly enriched in PMRG expression cluster A, such as renal cell carcinoma, ERBB signaling pathway, and endocytosis. The ERBB signaling pathway not only promotes cell proliferation but also directly creates an immunosuppressive TME to enable tumors to evade antitumor immune responses (61). The prognosis of PMRG expression cluster C was the worst, and metabolic pathways were mainly enriched, such as linoleic acid metabolism. GO analyses showed that the DEGs were significantly enriched in signal transduction and proliferation. KEGG analysis showed that DEGs were enriched in cancer metabolism and immune-related pathways, such as PI3K-Akt signaling pathway, PD-L1 expression and PD-1 checkpoint pathway in cancer. These results indicated that PMRG expression plays a key role in cancer and immune regulation.

On the basis of the prognostic DEGs, we developed the PGES and performed internal and external validation. PGES can be used as a reliable prognostic marker for patients with ccRCC. We found that cluster A has the lowest scores in the PMRG expression cluster and gene clusters, which can explain the better prognosis of cluster A. GSEA analysis showed that immune and metabolic pathways were significantly enriched in the high-PGES group. Given the close relationship between polyamines and immunity, we evaluated the immune cell infiltration of high- and low-PGES groups based on the ssGSEA, CIBERSORT, and ESTIMATE algorithms. The degree of immune cell infiltration increased in the high-PGES groups, especially T cells. T cell depletion markers were also highly expressed in the high-PGES groups, such as PDCD1, CTLA-4, TIGIT, LAG3, TNFRSF9, and CD27. Those markers can lead to T cell dysfunction (62–65). ccRCC mediates immune dysfunction by inducing immunosuppressive cells, such as Tregs, and inhibits the activities of the active molecules of effector T cells and antigen-presenting cells by upregulating checkpoints (66). Inhibitory receptors, immunosuppressive cytokines, and metabolic factors can promote T cell dysfunction in tumors (67). In our study, the high-PGES group had higher infiltration levels of T cells and expression of cytokines, but the prognosis was poor, suggesting that T cells in tumors are in a dysfunctional state. The immune and estimate scores of the high-PGES group were higher, indicating that the TME of the high-PGES group was more complex. Moreover, the TMB of the high-PGES group was higher. Theoretically, the number of neoantigens that can be recognized by T cells increases with TMB, and immunotherapy improves. However, T cells may be in a dysfunctional state, and the prognoses of patients with high TMB are poor, consistent with the previous study of ccRCC (68, 69). Tumor immune escape can better predict the prognosis of patients than TMB and PD-L1, and T cell dysfunction is an important cause of immune escape (70). The tumor formed an immunosuppressive microenvironment to promote immune escape (71). Impaired metabolism in the TME also contributes to immune escape (72). The high-PGES group had a higher level of immune escape and was positive correlated with Treg cells. Studies have shown that immune escape is mainly attributed to the high infiltration of Tregs cells and the expression of a large number of immunosuppressive receptors on T cells (73). PGES can effectively predict the effect of ccRCC on immunotherapy. In the IMvigor210 cohort, the PGES of nonresponders was higher, and the prognosis of patients in the high-PGES group was worse. Although the high-PGES patients had higher infiltration levels of immune cell, they may not be sensitive to immunotherapy due to immune escape.

SRM was a key PMRG identified by WGCNA. SRM is overexpressed in prostate cancer and can be used as a reliable biomarker and therapeutic target (74, 75). SRM overexpression can increase the drug resistance of bladder cancer to pirarubicin, and SRM knockdown can improve the chemotherapy efficacy of bladder cancer cells (76). We found that SRM still plays an oncogenic role in ccRCC, and SRM knockdown can inhibit malignant biological behavior of ccRCC cells. Compared with the control group, PGES decreased significantly after SRM knockdown, and SRM expression was positively correlated with PGES. ccRCC cells are resistant to sunitinib and sensitive to sorafenib after knockdown of SRM, which is consistent with our analysis that patients with high PGES are sensitive to sunitinib and patients with low PGES are sensitive to sorafenib. In addition, SRM can regulate the immune microenvironment, and SRM knockdown can inhibit the proliferation of fibroblasts (77). SRM knockdown can significantly reduce spermine level in ovarian cancer cells, and targeting polyamines can make ovarian cancer sensitive to immunotherapy (78). The regulatory mechanism of SRM in the ccRCC immune microenvironment deserves further study. Finally, we verified the expression of genes used to develop PGES in ccRCC tissues. EDA, SEMA3G, ENPP5, EMX2, and OPCML were favorable factors and had low expression levels in ccRCC tissues, whereas PCDHGC3 was a risk factor and highly expressed in cancer tissues.

Our study has some limitations. First, we only verified the expression of genes in the PGES. The applicability of PGES to a larger population should be further verified. Second, the PM mechanism in ccRCC immune microenvironment still needs further exploration. Third, the PGE score does not necessarily correlate with polyamine level, and the relationship with actual polyamine levels will require additional study.

## Conclusion

We revealed the biological characteristics of PMRG expression subtypes and developed the PGE score to accurately predict the prognoses of patients and response to immunotherapy. The key PM gene SRM can promote the malignant progression of ccRCC.

## Data availability statement

The original contributions presented in the study are included in the article/**Supplementary Material**. Further inquiries can be directed to the corresponding author.

## Ethics statement

The studies involving human participants were reviewed and approved by the ethics committee of Affiliated Haikou Hospital of Xiangya Medical College, Central South University. The patients/participants provided their written informed consent to participate in this study.

## Author contributions

MC designed the research. ZN, YG, and HC carried out the analyses. LZ and DH performed the experiments. SZ wrote the manuscript. All authors contributed to the article and approved the submitted version.

## Funding

The research is supported with fund from Hainan Provincial Natural Science Foundation of China (2017CXTD010), Finance science and technology project of Hainan province (ZDYF2019163 and ZDYF2021SHFZ249), the National Science Foundation of China (82160531 and 81760465), and the health department of Hainan province (19A200184).

## Conflict of interest

The authors declare that the research was conducted in the absence of any commercial or financial relationships that could be construed as a potential conflict of interest.

## Publisher’s note

All claims expressed in this article are solely those of the authors and do not necessarily represent those of their affiliated organizations, or those of the publisher, the editors and the reviewers. Any product that may be evaluated in this article, or claim that may be made by its manufacturer, is not guaranteed or endorsed by the publisher.
