# Supplementary figures and images for: Development of a polyamine gene expression score for predicting prognosis and treatment response in clear cell renal cell carcinoma
Source: Front Immunol. 2022 Nov 25;13:1048204. doi: 10.3389/fimmu.2022.1048204 (PMC9732944; doi:10.3389/fimmu.2022.1048204)

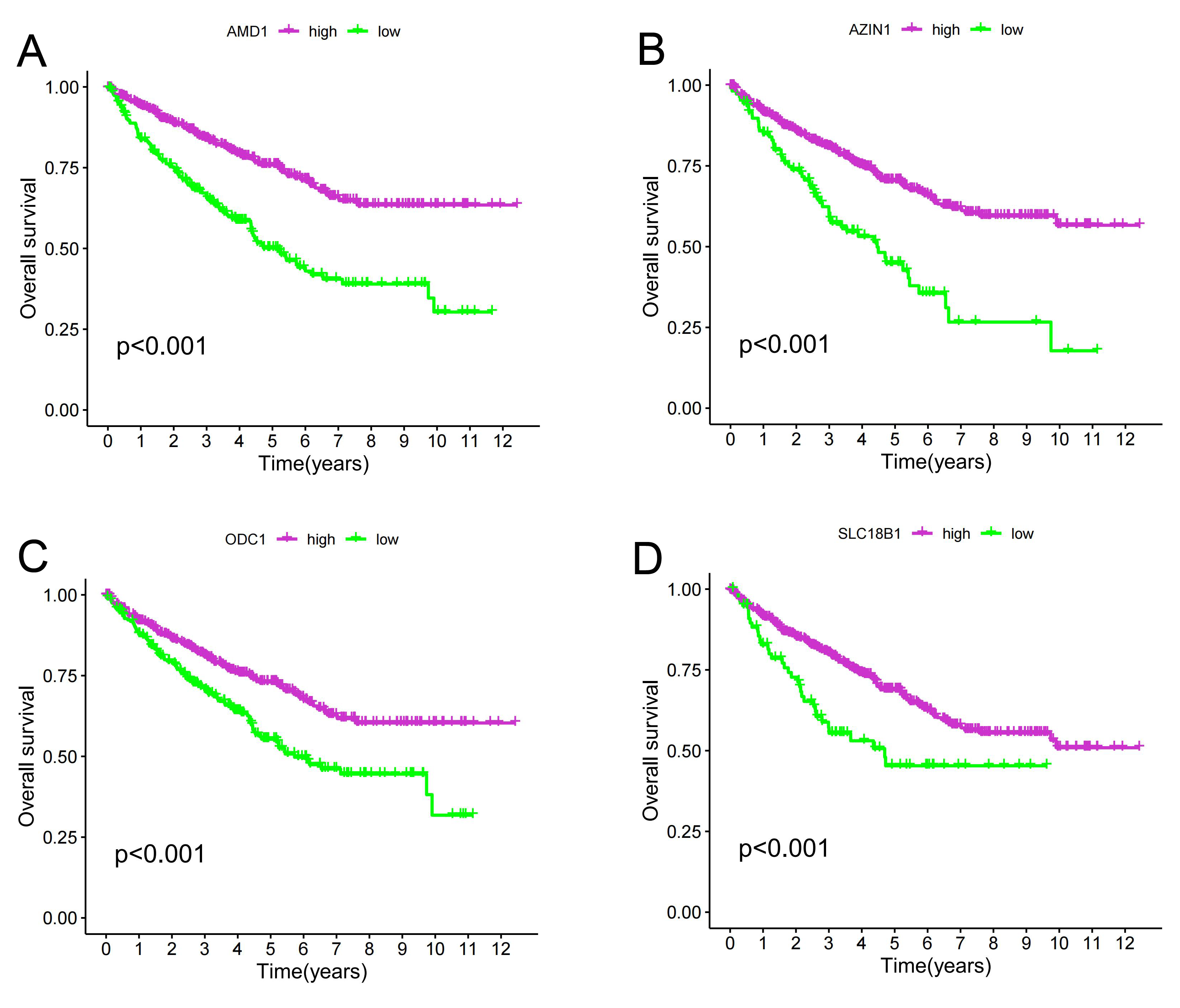

Supplement: Supplementary file 1 [file Image_1.tif]

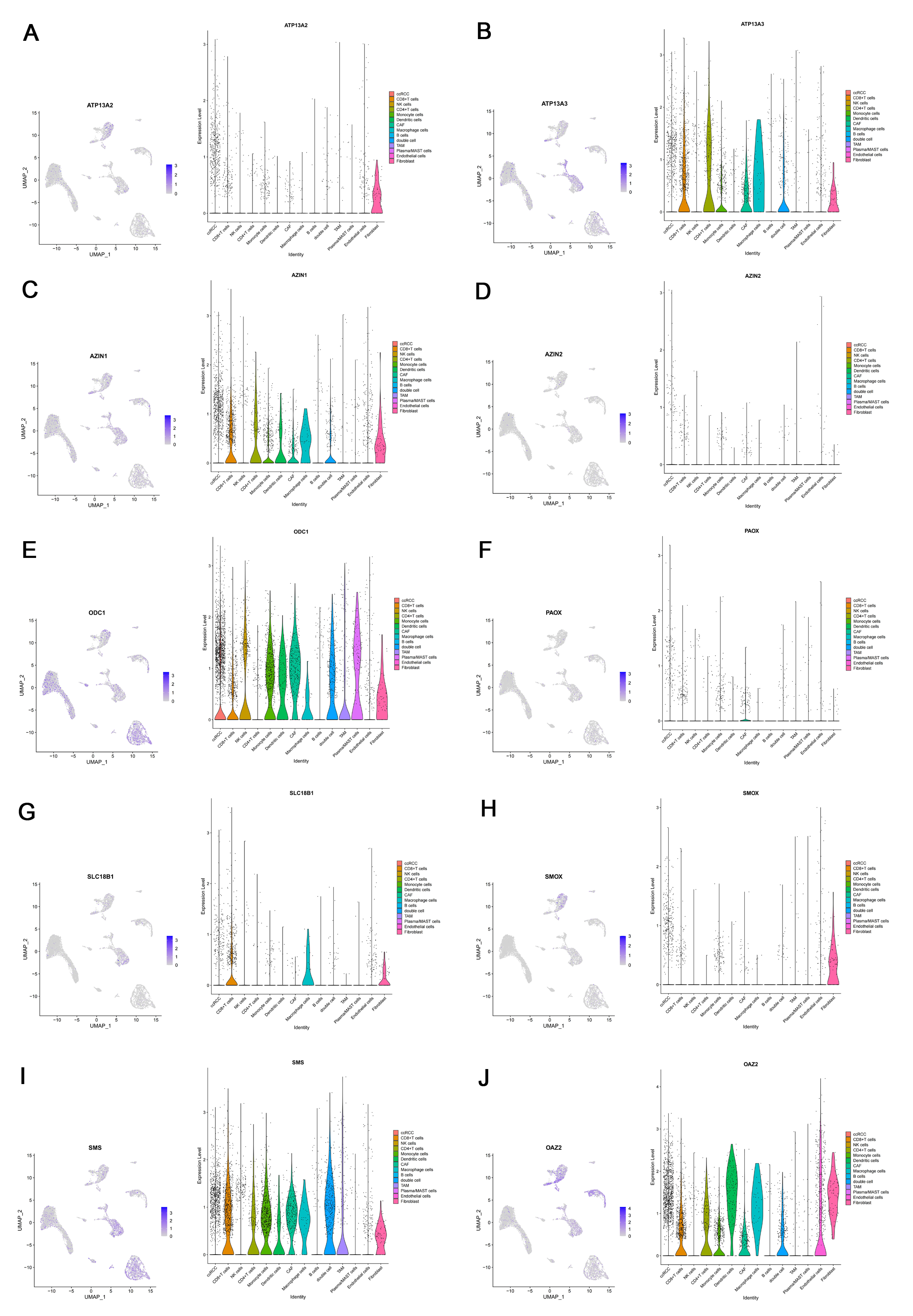

Supplement: Supplementary file 2 [file Image_2.tif]

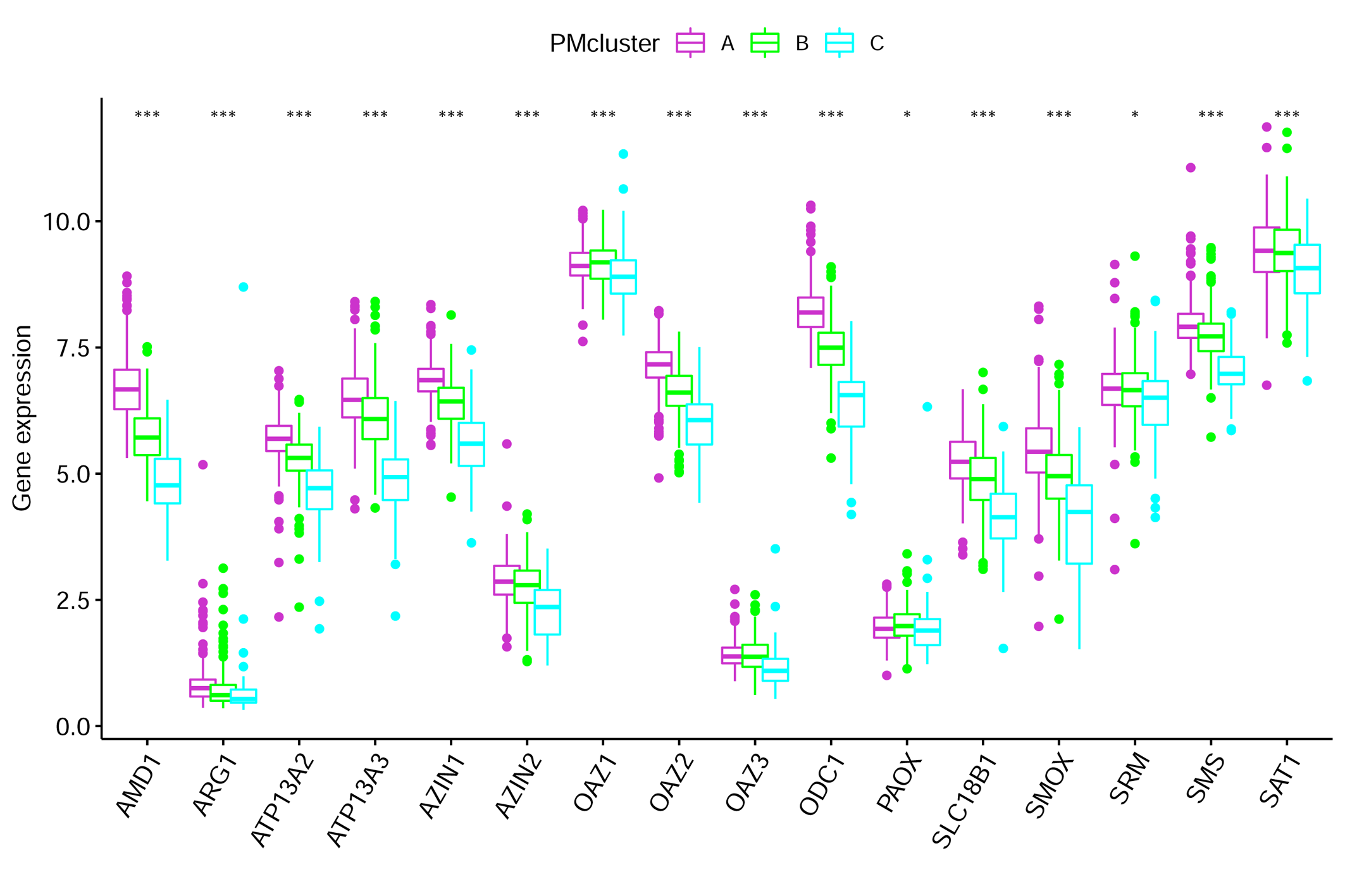

Supplement: Supplementary file 3 [file Image_3.tif]

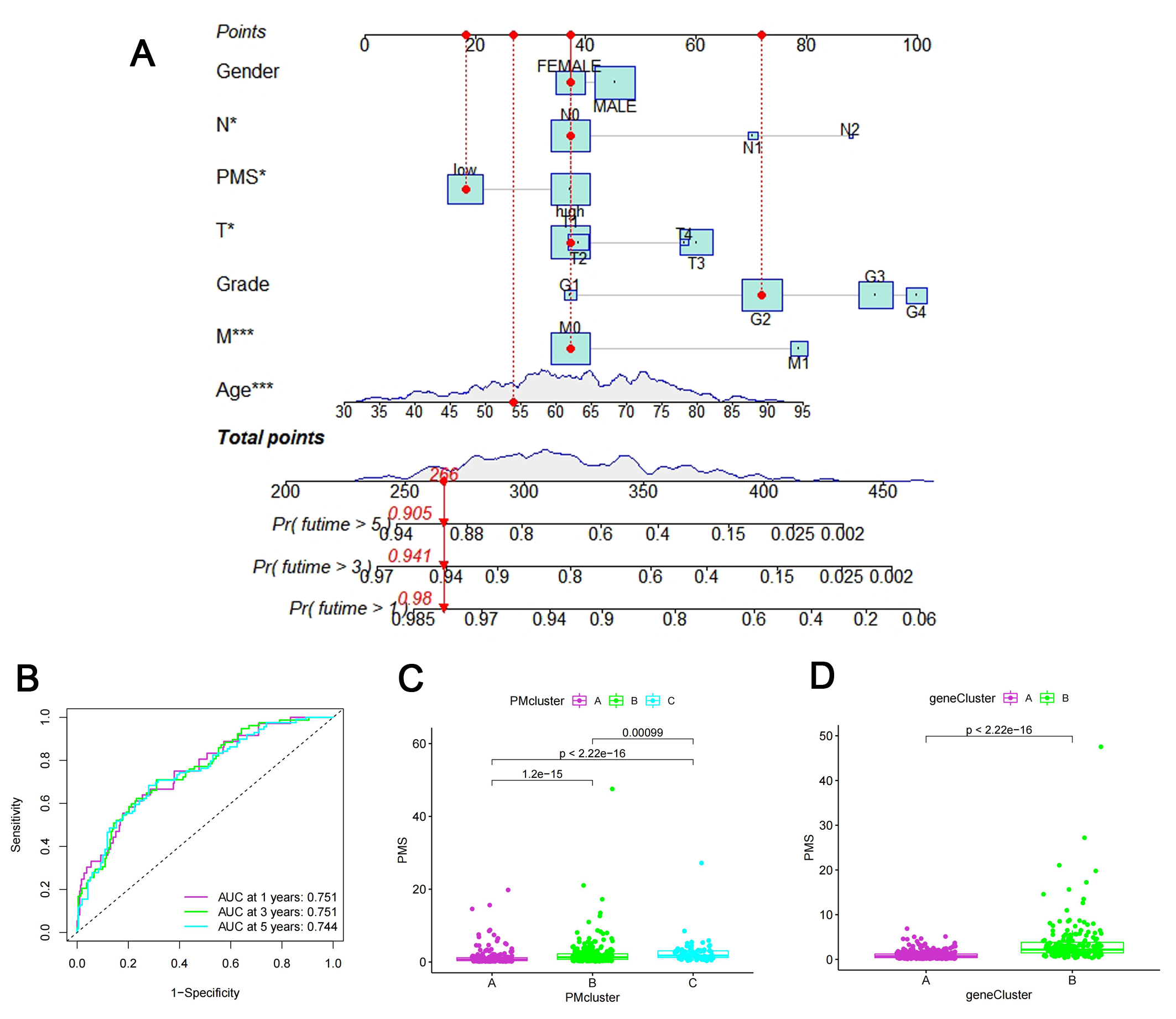

Supplement: Supplementary file 4 [file Image_4.tif]
