# Supplementary material for: Development of a polyamine gene expression score for predicting prognosis and treatment response in clear cell renal cell carcinoma
Source: Front Immunol. 2022 Nov 25;13:1048204. doi: 10.3389/fimmu.2022.1048204 (PMC9732944; doi:10.3389/fimmu.2022.1048204)
Supplement: Supplementary file 7 [file Table_3.docx]

**Supplementary Table S3. Primer sequences for qRT-PCR**

| Gene | Forward (5’-3’) | Reverse (5’-3’) |
| --- | --- | --- |
| GAPDH | CAGGAGGCATTGCTGATGAT | GAAGGCTGGGGCTCATTT |
| EDA | ATCTTTCAGGTGGAGTGCTCA | CACCACCTCATAGCTGGCAA |
| PCDHGC3 | TGTGGTCACAGTGTTCGGAG | CTCTGGGCCTGAGAGAAACG |
| SEMA3G | CTCCTACCGAGACCTCCTGT | AGTTGGCGCACTCTGTCAAA |
| ENPP5 | AATATCACCGCCATGCCACA | AATGCTGCCAAGAGAGACCC |
| EMX2 | TCAGCTACGCTAACTCCAGC | ACCATACTTTTACCTTGGAAGCG |
| OPCML | GTCAGTCAAGGAAGGCCAGG | ACTGGAATTCAGCCATGGGG |
| SRM | GATGATCGCCAACCTGCCTCTC | ATCTCACACTGGACCACGGACTC |
